# Sex-specific distribution of the uric acid-to-HDL cholesterol ratio and its association with arterial stiffness in a Japanese health check-up population: a cross-sectional analysis

**DOI:** 10.3389/fendo.2026.1847114

**Published:** 2026-05-21

**Authors:** Yongsheng Qu, Yaohui Jiang

**Affiliations:** Department of Cardiac Intensive Care Unit, Fuwai Central China Cardiovascular Hospital, Central China Fuwai Hospital of Zhengzhou University, Zhengzhou, Henan, China

**Keywords:** arterial stiffness, brachial-ankle pulse wave velocity, cross-sectional study, Japanese population, uric acid to HDL-cholesterol ratio

## Abstract

**Background:**

The uric acid to HDL-cholesterol ratio (UHR) is a composite marker of metabolic and inflammatory imbalance. Although a previous study in a Japanese health check-up population reported a positive and nonlinear association between UHR and brachial-ankle pulse wave velocity (baPWV), the interpretation of sex-specific heterogeneity remains unclear. Therefore, the association between UHR and baPWV was re-examined, with particular attention to the sex-specific distribution of UHR and the robustness of sex-stratified findings.

**Methods:**

This cross-sectional analysis was based on 912 participants. UHR was derived from the ratio of serum uric acid to HDL-cholesterol and was modeled as both a continuous measure and a quartile-based variable. Multivariable linear regression was used to assess its association with baPWV. To further characterize the shape of the association, restricted cubic spline fitting and piecewise linear regression were performed. Stratified analyses were also conducted across subgroups defined by age, sex, estimated glomerular filtration rate, drinking behavior, smoking, and physical activity, with additional attention to the distribution of UHR in men and women and menopausal status among women.

**Results:**

baPWV increased across UHR quartiles. After multivariable adjustment, UHR remained positively associated with baPWV, and higher UHR quartiles showed significantly higher baPWV than the lowest quartile, with a significant trend. Restricted cubic spline analysis showed a nonlinear association between UHR and baPWV (P for overall of 0.001; P for non-linearity of 0.002). The inflection point was identified at a UHR value of 6.261, with a significant positive association below this point but no statistically significant association above it. Subgroup analyses suggested possible heterogeneity by age and sex. The association was statistically clear among participants younger than 65 years and among women, whereas the estimate in older participants was imprecise and no significant association was observed in men.

**Conclusion:**

UHR was positively associated with baPWV, with evidence of a nonlinear pattern and an inflection point at a UHR value of 6.261. The sex-specific findings should be interpreted cautiously because UHR values differed markedly between men and women and women were sparsely represented in the higher UHR range. UHR may help identify medication-free individuals with higher baPWV, but prospective studies are needed to confirm its incremental clinical value.

## 1 Introduction

Cardiovascular disease (CVD) remains a major cause of mortality and disability worldwide, placing a heavy burden on public health (1, 2). Because clinical cardiovascular events are often preceded by a prolonged asymptomatic phase of vascular injury, early identification of subclinical vascular abnormalities and appropriate risk stratification are essential for primary prevention (3). Arterial stiffness is widely recognized as an important manifestation of subclinical vascular damage (1). In addition to its role in the development of CVD, it has also been shown to predict future cardiovascular events and all-cause mortality independently (4, 5). Therefore, identifying individuals with increased arterial stiffness in clinical or health check-up settings is of clear clinical importance.

Brachial-ankle pulse wave velocity (baPWV) is one of the most commonly used noninvasive indices for the assessment of arterial stiffness (6). Owing to its relative simplicity, good reproducibility, and suitability for large-scale screening, baPWV has been extensively applied in health examination settings and epidemiological studies, particularly in East Asian populations (7, 8). In Japan, baPWV has also been widely used to evaluate subclinical target organ damage and has become a routine component of health check-up programs (9). Nevertheless, baPWV measurement still requires dedicated equipment and trained personnel (10–12). Thus, there remains a practical need to identify simple and easily accessible serum biomarkers derived from routine laboratory testing that are associated with higher baPWV or greater arterial stiffness in health check-up settings.

Uric acid (UA) and high-density lipoprotein cholesterol (HDL-C) are two routinely measured biochemical markers that have been widely studied in relation to vascular function. Earlier studies have indicated that increased UA levels are associated with biological processes relevant to vascular stiffness, such as oxidative stress, persistent inflammation, endothelial impairment, and vascular smooth muscle cell growth (13–16). By contrast, HDL-C has been associated with reverse cholesterol transport, anti-inflammatory, antioxidant, and endothelial-supportive effects, and endothelial homeostasis (17–19), whereas reduced HDL-C levels have been associated with greater arterial stiffness (20, 21). However, either marker alone reflects only one aspect of the complex pathophysiological processes underlying vascular injury and cardiometabolic imbalance.

Against this background, UHR has recently attracted increasing interest as a composite biomarker. Compared with UA or HDL-C alone, UHR combines information from elevated UA and lower HDL-C, and may therefore provide a composite measure associated with cardiometabolic disturbance. Accumulating evidence suggests that elevated UHR is related not only to adverse cardiovascular outcomes but also to metabolic disturbances, including type 2 diabetes, insulin resistance, and metabolic syndrome (22, 23). These findings support its potential role in cardiovascular risk assessment.

Evidence regarding the relationship between UHR and arterial stiffness remains limited but is emerging. Wang et al. previously reported a nonlinear association between UHR and baPWV in a Japanese health check-up population, identified an inflection point, and suggested that the association differed across subgroups, particularly by sex (24). Therefore, the present study does not aim to claim the first description of the UHR-baPWV association in this population. Instead, it re-examines this association with a narrower focus on the interpretation of sex-specific heterogeneity. In particular, because UHR distributions differ markedly between men and women, and because sparse data in the higher UHR range among women may affect subgroup estimates, the present analysis further evaluated sex-specific UHR distribution, sex composition across UHR categories, sequential sex-stratified models, and menopausal-status-adjusted estimates among women.

## 2 Materials and methods

### 2.1 Study population and data source

This analysis was performed using an open-access dataset and followed a cross-sectional design. The data originated from a routine health screening program conducted at Murakami Memorial Hospital Medical Health Checkup Center, Japan (25). The screening period extended from March 2004 to December 2012. Ethical approval and written informed consent had already been obtained in the original survey (25). Since only anonymized records were used here, no further contact with participants or additional procedures were required.

### 2.2 Eligibility criteria

The source dataset initially contained 1,445 individuals (25). Participant selection was then refined according to prespecified criteria. Exclusion criteria included current medication use, pregnancy, exposure to exogenous hormones such as oral contraceptives or hormone replacement therapy, and serological evidence of hepatitis B or hepatitis C infection. The exclusion of participants using medications was intended to reduce potential confounding from drug treatments that may influence serum uric acid, lipid profiles, blood pressure, glucose metabolism, or vascular measurements. Participants with ABI values below 0.95 were also removed to reduce the potential influence of peripheral arterial disease on arterial stiffness assessment (26). After screening, 912 subjects were retained for the final analysis (25).

### 2.3 Clinical variables and exposure assessment

The health checkup records provided information on demographic characteristics, lifestyle factors, physical measurements, and blood biochemistry (27, 28). Age, sex, smoking, alcohol use, and exercise habits were collected from standardized questionnaires. During the examination, height and weight were measured, and BMI was determined using the standard formula of weight (kg)/height^2^ (m^2^).

After an overnight fast, venous blood was collected for biochemical analysis. The laboratory variables used in this study included AST, ALT, GGT, fasting plasma glucose, UA, LDL-C, HDL-C, triglycerides, and total cholesterol. Kidney function was represented by eGFR (29).

The exposure of interest was UHR, calculated by dividing serum uric acid by HDL-C. For statistical analysis, UHR was handled in two forms: as a continuous measurement and as a quartile-based categorical variable.

The presence of fatty liver was assessed by abdominal ultrasound examination (30, 31). In the original screening setting, experienced physicians made this judgment according to typical sonographic findings such as increased hepatic echogenicity, liver-kidney contrast, posterior attenuation, and poor visualization of intrahepatic vascular margins. Smoking status was categorized as current versus non-current smoking. Alcohol intake was defined from recent drinking information, and regular exercise referred to physical activity performed at least once each week.

### 2.4 Measurement of arterial stiffness

Arterial stiffness was represented by baPWV, and ABI was measured simultaneously. Both indices were obtained using an automated waveform analysis system. Before measurement, each participant rested quietly in the supine position for no less than 5 minutes. The device then recorded blood pressure and pulse wave signals from the extremities and generated baPWV values automatically from transit time and estimated vascular path length. Because an abnormally low ABI may interfere with baPWV interpretation, individuals with ABI <0.95 had been excluded before analysis (32).

### 2.5 Statistical analysis

RStudio was used for the statistical analyses. Continuous variables are expressed as mean ± standard deviation or median with interquartile range according to their distribution, whereas categorical variables are summarized as counts and percentages. Group differences across UHR quartiles were assessed by one-way analysis of variance, Kruskal-Wallis testing, or the chi-square test, as appropriate.

The association between UHR and baPWV was examined by linear regression. In these models, baPWV served as the dependent variable, whereas UHR was entered either continuously or by quartile. Several adjustment schemes were applied: an unadjusted model; Model 1 including age, sex, and BMI; Model 2 additionally including ALT, AST, GGT, ABI, TC, and LDL-C; and Model 3 further incorporating alcohol use and smoking status. To assess trend across quartiles, the ordered quartile categories were entered into the regression models as a ranked variable.

Because a linear assumption might be inappropriate, restricted cubic splines were fitted to explore the shape of the UHR-baPWV relationship. A two-segment linear model was then used to evaluate whether a turning point was present. The likelihood ratio test was applied to compare this model with the ordinary linear model.

To determine whether the association differed across participant characteristics, subgroup analyses were conducted according to age, sex, eGFR, drinking status, smoking status, and exercise habits. Interaction testing was used to assess heterogeneity between strata. In light of the sex-specific pattern reported by Wang et al. (24), additional analyses were conducted to facilitate the interpretation of sex-related heterogeneity. These analyses included comparison of the UHR distribution between men and women, assessment of the numbers of men and women across overall UHR quartiles, sex-stratified regression models, and a female-specific model with additional adjustment for menopausal status. All statistical tests were two-sided, and P values below 0.05 were considered statistically significant.

## 3 Results

### 3.1 Participant profiles by UHR quartile group

Overall, 912 individuals were analyzed and stratified into four equally sized groups according to UHR quartiles. The corresponding baseline data are provided in **Table 1**. Mean baPWV values increased across the quartiles of UHR, measuring 1365.71 ± 218.13 in Q1, 1401.63 ± 226.76 in Q2, 1426.25 ± 214.35 in Q3, and 1469.41 ± 304.46 in Q4, with a statistically significant overall difference (P < 0.001).

**Table 1:** Baseline characteristic of the study population according to UHR.

Variables	Total (n = 912)	Q1 (n = 228)	Q2 (n = 228)	Q3 (n = 228)	Q4 (n = 228)	P value
baPWV	1415.75 ± 246.25	1365.71 ± 218.13	1401.63 ± 226.76	1426.25 ± 214.35	1469.41 ± 304.46	*< 0.001*
Sex, n (%)						*< 0.001*
Male	592 (64.91)	37 (16.23)	138 (60.53)	195 (85.53)	222 (97.37)	
Female	320 (35.09)	191 (83.77)	90 (39.47)	33 (14.47)	6 (2.63)	
Age, (years)	51.13 ± 9.57	51.83 ± 9.28	51.51 ± 9.26	50.62 ± 9.78	50.57 ± 9.95	0.392
BMI, (kg/m2)	23.13 ± 3.12	21.47 ± 2.59	22.50 ± 2.71	23.92 ± 2.85	24.62 ± 3.31	*< 0.001*
SBP, (mmHg)	120.25 ± 14.96	114.06 ± 13.98	119.29 ± 14.65	123.54 ± 14.19	124.09 ± 14.89	*< 0.001*
DBP, (mmHg)	76.14 ± 10.01	70.77 ± 9.07	75.37 ± 9.66	78.58 ± 9.14	79.84 ± 9.70	*< 0.001*
AST, (IU/L)	20.85 ± 8.09	18.64 ± 5.49	20.93 ± 9.79	21.12 ± 7.42	22.72 ± 8.54	*< 0.001*
ALT, (IU/L)	22.69 ± 14.29	16.07 ± 5.94	21.42 ± 15.20	23.57 ± 12.62	29.68 ± 17.28	*< 0.001*
GGT, (IU/L)	19.00 (14.00, 28.00)	13.00 (11.00, 17.00)	17.00 (13.00, 30.00)	20.50 (16.00, 30.25)	23.00 (18.00, 34.00)	*< 0.001*
FPG, (mg/dl)	98.05 ± 14.06	94.39 ± 18.56	97.95 ± 12.74	99.04 ± 11.97	100.81 ± 11.02	*< 0.001*
UA, (mg/dl)	5.26 ± 1.38	3.79 ± 0.84	4.93 ± 0.88	5.70 ± 0.85	6.61 ± 1.05	*< 0.001*
TC, (mg/dl)	209.82 ± 35.97	212.00 ± 35.41	212.06 ± 36.06	206.78 ± 36.55	208.44 ± 35.80	0.299
TG, (mg/dl)	81.00 (53.00, 124.00)	53.00 (38.00, 70.25)	73.00 (51.75, 108.50)	84.00 (61.00, 116.25)	138.00 (100.00, 191.25)	*< 0.001*
HDL-c, (mg/dl)	53.54 ± 14.60	68.82 ± 13.45	57.40 ± 9.74	49.12 ± 7.44	38.81 ± 6.06	*< 0.001*
LDL-c, (mg/dl)	128.06 ± 31.69	120.63 ± 31.08	128.46 ± 31.13	130.17 ± 31.35	132.98 ± 32.05	*< 0.001*
eGFR, (mL/min/1.73 m2)	70.41 ± 12.04	74.45 ± 13.11	71.18 ± 11.22	69.40 ± 10.75	66.62 ± 11.66	*< 0.001*
ABI	1.19 (1.14, 1.24)	1.16 (1.12, 1.21)	1.18 (1.13, 1.22)	1.20 (1.16, 1.25)	1.20 (1.15, 1.25)	*< 0.001*
Alcohol consumption, n (%)						*< 0.001*
None or minimal	595 (65.24)	189 (82.89)	144 (63.16)	120 (52.63)	142 (62.28)	
Light	149 (16.34)	22 (9.65)	38 (16.67)	52 (22.81)	37 (16.23)	
Moderate	88 (9.65)	9 (3.95)	25 (10.96)	32 (14.04)	22 (9.65)	
Heavy	80 (8.77)	8 (3.51)	21 (9.21)	24 (10.53)	27 (11.84)	
Smoking status, n (%)						*< 0.001*
None or Past	715 (78.40)	212 (92.98)	178 (78.07)	173 (75.88)	152 (66.67)	
Current	197 (21.60)	16 (7.02)	50 (21.93)	55 (24.12)	76 (33.33)	
Habit of exercise, n (%)						*0.002*
No	719 (80.25)	160 (72.4)	177 (78.67)	190 (84.44)	192 (85.33)	
Yes	177 (19.75)	61 (27.6)	48 (21.33)	35 (15.56)	33 (14.67)	
Fatty liver, n (%)						*< 0.001*
No	646 (70.91)	212 (92.98)	183 (80.62)	153 (67.11)	98 (42.98)	
Yes	265 (29.09)	16 (7.02)	44 (19.38)	75 (32.89)	130 (57.02)	

BaPWV, brachial-ankle pulse wave velocity; 95% CI, 95% confidence interval; UHR, uric acid to high-density lipoprotein cholesterol ratio; BMI, body mass index; SBP, systolic blood pressure; DBP, diastolic blood pressure; AST, aspartate aminotransferase; ALT, alanine aminotransferase; GGT, γ-glutamyltranspeptidase; FPG, fasting plasma glucose; UA, uric acid; TC, total cholesterol; TG, triglyceride; HDL-c, high‐density lipoprotein cholesterol; LDL-C, low-density lipoprotein cholesterol; eGFR, estimated glomerular filtration rate; ABI, ankle-brachial index.

Across increasing UHR quartiles, participants were more likely to be male and to exhibit a less favorable cardiometabolic profile, characterized by higher BMI, blood pressure, liver enzyme levels, fasting glucose, uric acid, triglycerides, LDL-C, and ABI, together with lower HDL-C and eGFR (all P < 0.05). Drinking status, smoking status, exercise habits, and fatty liver prevalence also varied among the groups (all P < 0.05). By comparison, neither age nor total cholesterol showed a significant difference across UHR quartiles (all P > 0.05).

### 3.2 Regression analyses of the relationship between UHR and baPWV

In the univariate linear regression analysis, UHR was significantly associated with baPWV in a positive direction (β of 16.96, 95% CI from 8.23 to 25.69, P < 0.001; **Supplementary Table 1**).

**Table 2** summarizes the multivariable regression results. With UHR entered as a continuous variable, each 1-unit increase in UHR was associated with a 16.96-unit increase in baPWV in the crude model (95% CI from 8.25 to 25.68, P < 0.001). The positive association remained significant after adjustment for age, sex, and BMI (β of 12.92, 95% CI from 3.23 to 22.60, P of 0.009). Additional adjustment for ALT, AST, GGT, ABI, TC, and LDL-c (Model 2) did not materially alter the result (β of 11.85, 95% CI from 1.60 to 22.10, P of 0.024). The association persisted even after further controlling for drinking and smoking status in Model 3 (β of 10.93, 95% CI from 0.64 to 21.21, P of 0.038).

**Table 2:** Multivariable-adjust β and 95%CI of the UHR index associated with baPWV.

Variable	Unadjusted		Model 1		Model 2		Model 3	
	β (95% CI)	P value	β (95% CI)	P value	β (95% CI)	P value	β (95% CI)	P value
UHR	16.96 (8.25~25.68)	<0.001	12.92 (3.23~22.60)	0.009	11.85 (1.60~22.10)	0.024	10.93 (0.64~21.21)	0.038
1st Quartile(≤2.74)	Ref		Ref		Ref		Ref	
2nd Quartile (2.74-3.90)	35.91 (-8.83~80.66)	0.116	23.42 (-18.55~65.38)	0.274	27.74 (-15.56~71.04)	0.210	28.22 (-15.23~71.66)	0.203
3rd Quartile (3.90-5.20)	60.53 (15.79~105.27)	0.008	47.19 (-0.46~94.84)	0.053	56.09 (5.57~106.62)	0.030	58.47 (7.68~109.25)	0.024
4th Quartile (≥5.20)	103.7 (58.95~148.44)	<0.001	84.83 (33.74~135.93)	0.001	87.63 (32.67~142.59)	0.002	85.28 (30.15~140.40)	0.002
*P* for trend		<0.001		0.001		0.001		0.002

Model 1 adjust for age, sex, BMI.

Model 2 adjust for Model 1+ ALT, AST, GGT, ABI, TC, LDL-c.

Model 3 adjust for Model 1+ Model 2 + alcohol consumption, smoking status.

UHR, uric acid to high-density lipoprotein cholesterol ratio; baPWV, brachial-ankle pulse wave velocity; 95% CI,95% confidence interval; Ref, reference; BMI, body mass index; ALT, alanie aminotransferase; AST, aspartate aminotransferase; LDL-c, low-density lipoprotein cholesterol; TC, total cholesterol; GGT, gamma-glutamyltransferase; ABI, ankle-brachial index.

In the quartile-based analysis, and taking Q1 as the reference group, baPWV was significantly higher in Q3 and Q4 after full adjustment, with estimated differences of 58.47 (95% CI from 7.68 to 109.25, P of 0.024) and 85.28 (95% CI from 30.15 to 140.40, P of 0.002), respectively. No significant difference was observed between Q2 and Q1 (β of 28.22, 95% CI from -15.23 to 71.66, P of 0.203). In addition, a significant trend was also observed across increasing UHR quartiles (P for trend of 0.002).

### 3.3 Nonlinear association and threshold pattern between UHR and baPWV

**Figure 1:**
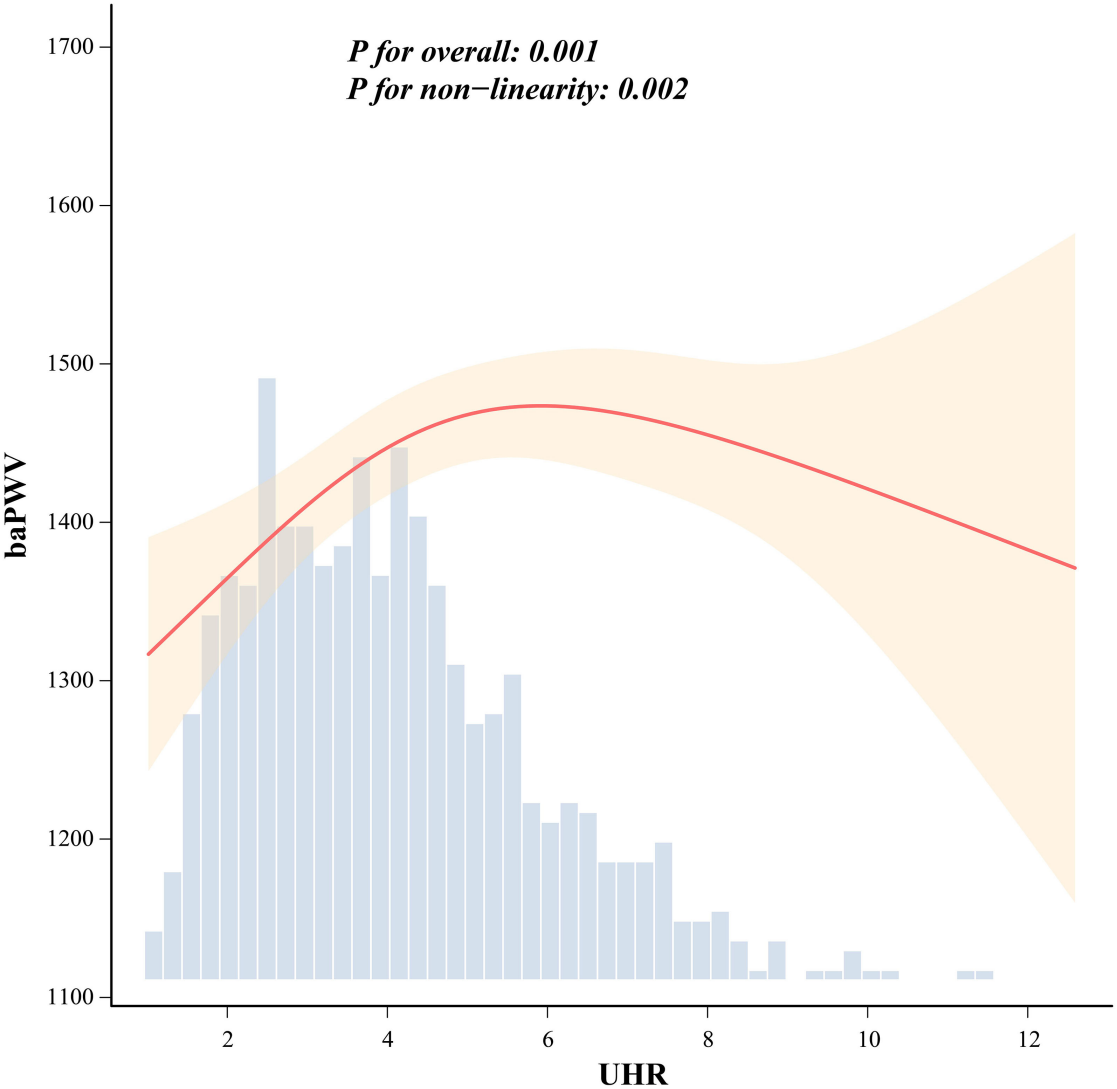
Nonlinear association between UHR and arterial stiffness (baPWV) assessed by restricted cubic spline analysis. Restricted cubic spline showing the association between uric acid-to-HDL cholesterol ratio (UHR) and brachial-ankle pulse wave velocity (baPWV). The solid red line represents the estimated association, and the shaded area indicates the 95% confidence interval. The histogram shows the distribution of UHR. P for overall = 0.001; P for non-linearity = 0.002.

The restricted cubic spline analysis is illustrated in **Figure 1**. Following adjustment for potential confounders, the overall UHR–baPWV association was still statistically significant (P for overall of 0.001), together with evidence of nonlinearity (P for non-linearity of 0.002). The curve suggested that baPWV tended to be higher at increasing UHR values, although the slope became less steep at higher UHR values.

To further characterize this pattern, a two-piecewise linear regression model was applied (**Table 3**). The inflection point was identified at a UHR value of 6.261. Compared with the conventional linear model, the piecewise model showed a better fit (P for likelihood ratio test of 0.024). At UHR values below 6.261, UHR was positively associated with baPWV (β of 41.845, 95% CI from 25.584 to 58.107, P < 0.001). When UHR was 6.261 or higher, the association was no longer statistically significant (β of -21.886, 95% CI from -76.032 to 32.260, P of 0.425). These findings suggest an exploratory threshold pattern in the cross-sectional association between UHR and baPWV.

**Table 3:** Threshold effect analysis of UHR index associated with baPWV using two-piecewise linear regression.

Outcome	baPWV	
	Adjusted β (95% CI)	*P*-value
Inflection point of UHR	6.261	
UHR < 6.261	41.845 (25.584, 58.107)	< 0.001
UHR ≥ 6.261	-21.886 (-76.032, 32.260)	0.425
*P* for Likelihood Ratio test	0.024	
Non-linear Test*1	<0.001	
Non-linear Test*2	0.001	

Adjusted for age, sex, BMI, ALT, AST, GGT, ABI, TC, LDL-c, alcohol consumption, smoking status.

Albumin; UHR, uric acid to high-density lipoprotein cholesterol ratio; baPWV, brachial-ankle pulse wave velocity; 95% CI,95% confidence interval; Ref, reference; BMI, body mass index; ALT, alanie aminotransferase; AST, aspartate aminotransferase; LDL-c, low-density lipoprotein cholesterol; TC, total cholesterol; GGT, gamma-glutamyltransferase; ABI, ankle-brachial index.

### 3.4 Subgroup analyses

**Figure 2:**
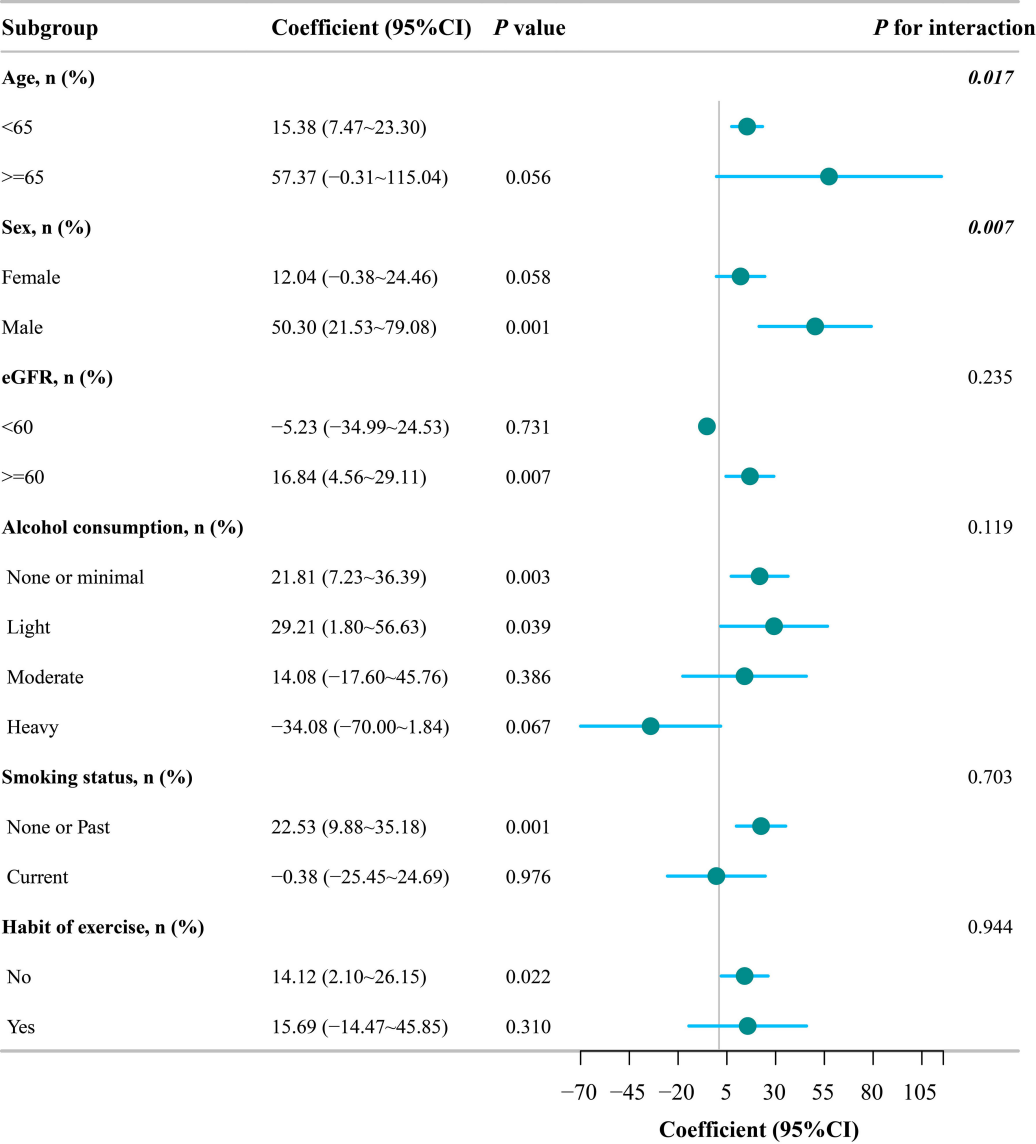
Subgroup analyses of the association between UHR and baPWV. Forest plot showing the association between uric acid-to-HDL cholesterol ratio (UHR) and brachial-ankle pulse wave velocity (baPWV) across subgroups stratified by age, sex, eGFR, alcohol consumption, smoking status, and exercise habits. Data are presented as regression coefficients with 95% confidence intervals.

Subgroup analyses are presented in **Figure 2**. Evidence of interaction was observed for age and sex, whereas no significant interactions were found for eGFR, alcohol consumption, smoking status, or exercise habits.

In the age-stratified analysis, UHR was positively associated with baPWV among participants younger than 65 years (β of 15.38, 95% CI from 7.47 to 23.30). Among participants aged 65 years or older, the point estimate was numerically larger but less precise, with a wide confidence interval crossing the null value (β of 57.37, 95% CI from -0.31 to 115.04, P of 0.056). The interaction by age was statistically significant (P for interaction of 0.017). These results indicate that the association was statistically clear in participants younger than 65 years, but they do not support the interpretation that the association was stronger in this subgroup.

Because a significant interaction between UHR and sex was observed, we further examined the sex-specific distribution of UHR and the sex-stratified association between UHR and baPWV. UHR values differed markedly between men and women. Men had higher and more widely distributed UHR values than women [median (IQR): 4.60 (3.75 to 5.89) vs. 2.58 (2.04 to 3.21), P < 0.001; **Supplementary Table 2**]. The sex-specific distribution of UHR is further illustrated in **Supplementary Figure 1**. Across the overall UHR quartiles, the numbers of men and women were 37/191 in Q1, 138/90 in Q2, 195/33 in Q3, and 222/6 in Q4, respectively. These data indicate that the upper end of the overall UHR distribution was dominated by men and that women contributed few observations to the high-UHR range.

In sex-stratified regression analyses, UHR was not significantly associated with baPWV in men in the fully adjusted model (β = 5.46, 95% CI: -5.94 to 16.85, P = 0.348), whereas a positive association was observed in women (β = 46.40, 95% CI: 19.48 to 73.32, P = 0.001). The interaction between UHR and sex remained statistically significant in the fully adjusted model (P for interaction = 0.003). After additional adjustment for menopausal status in women, the association remained significant (β = 46.56, 95% CI: 19.60 to 73.51, P = 0.001; **Supplementary Table 3**).

Stratified analyses by eGFR, drinking status, smoking status, and exercise habits showed no significant interactions (all P for interaction > 0.05), suggesting that the association between UHR and baPWV was generally consistent across these subgroups.

## 4 Discussion

This study examined the cross-sectional association between UHR and arterial stiffness, as measured by baPWV, in a Japanese health check-up population. Higher UHR was associated with higher baPWV, and this association persisted after adjustment for multiple covariates. Beyond the main effect, the data also suggested a nonlinear pattern, with the association mainly observed below the identified inflection point rather than across the entire UHR distribution. In addition, the strength of the association was not uniform across subgroups, showing some variation by age and sex. Overall, these findings indicate that UHR is associated with higher baPWV in this cross-sectional health check-up population, but they do not establish a temporal or causal relationship. Importantly, given the previous work by Wang et al. using the same source population, the contribution of the present analysis is mainly the focused evaluation and cautious interpretation of sex-specific patterns rather than identification of a wholly new population-level association.

In recent years, growing attention has been directed toward composite biomarkers in risk prediction because they can capture information from multiple pathophysiological pathways within a single index (17). A well-known example is the triglyceride-to-HDL-C ratio, which has been shown to outperform triglycerides or HDL-C alone in identifying insulin resistance, diabetes, and cardiovascular disease risk (21). UHR is based on a similar concept, integrating information related to both purine metabolism and lipid abnormalities. A number of studies have suggested that UHR may provide better risk stratification than either uric acid or HDL-C alone in metabolic syndrome, type 2 diabetes, and cardiovascular disease. In an Iranian population, Yazdi et al. reported that UHR had greater diagnostic value for metabolic syndrome than either uric acid or HDL-C individually, with an area under the curve of 0.78, indicating a good ability to identify individuals at high metabolic risk (23). A longitudinal study by Bazmandegan et al. further showed that baseline UHR independently predicted the subsequent development of metabolic syndrome in patients with type 2 diabetes (22). In the field of coronary artery disease, Aktaş et al. found that UHR was positively associated with the coronary SYNTAX score and independently reflected lesion severity (17). By comparison, much less is known about the relationship between UHR and subclinical vascular abnormalities such as arterial stiffness. Wang et al. provided important evidence by showing a nonlinear UHR-baPWV association in a Japanese health check-up population (24). The present analysis is consistent with their work in showing a positive and nonlinear UHR–baPWV association. However, its added value lies in extending the interpretation of sex-specific heterogeneity by reporting the distribution of UHR separately in men and women, the sex composition across UHR quartiles, sequentially adjusted sex-stratified regression models, and an additional female-specific model adjusted for menopausal status. These additional analyses suggest that the sex-specific pattern should be interpreted cautiously because UHR values were markedly different between men and women and women were sparsely represented in the higher UHR range.

The observed association between UHR and baPWV can be interpreted in the context of previous biological and epidemiological evidence, although the present study cannot establish mechanism. UHR combines two routinely measured markers with different vascular implications. Uric acid has been associated with oxidative stress, low-grade inflammation, endothelial dysfunction, and vascular smooth muscle cell activation, all of which are involved in arterial wall remodeling (9, 33). By contrast, HDL-C is generally regarded as protective because it is related to antioxidant activity, anti-inflammatory effects, and maintenance of endothelial homeostasis (17, 34). A higher UHR may therefore represent a composite biochemical profile characterized by higher UA and lower HDL-C. This interpretation is consistent with the observed association between higher UHR and higher baPWV, but the cross-sectional design does not allow mechanistic inference.

Another point worth noting is that the observed association was not linear. The spline and piecewise analyses suggested that increases in UHR were more clearly associated with baPWV at relatively lower levels, whereas the slope became flatter at higher values. This nonlinear pattern should be interpreted as a description of the cross-sectional association rather than evidence of a biological threshold or disease progression. The spline and piecewise analyses suggested that the association between UHR and baPWV was more apparent at lower-to-middle UHR values, whereas the slope became flatter at higher values. This flattening may reflect heterogeneity in participant characteristics, sparse data at the upper end of the UHR distribution, or residual confounding. Therefore, the threshold should be regarded as exploratory and requires confirmation in prospective studies.

The subgroup findings, particularly those stratified by age and sex, should be interpreted with caution. For age, the association reached statistical significance only in participants younger than 65 years; however, this should not be interpreted as evidence of a stronger association in younger individuals. The estimate among older participants was less precise, and its wide confidence interval indicates considerable uncertainty. Therefore, the observed age interaction is better viewed as an exploratory signal that requires confirmation rather than definitive evidence of age-dependent effect modification.

The sex-stratified findings require cautious interpretation. Wang et al. reported that UHR and baPWV were significantly higher in men than in women and that a significantly positive correlation between UHR and baPWV was observed only in women (24). Our findings were directionally consistent with this pattern: UHR values were substantially higher and more widely distributed in men than in women, whereas the association between UHR and baPWV was statistically evident in women but not in men in the fully adjusted model. However, this pattern should not be interpreted simply as evidence that UHR is biologically relevant only in women. The distribution of overall UHR quartiles was markedly imbalanced by sex, and only six women were included in the highest overall UHR quartile. This sparse representation of women in the higher UHR range may reduce the stability of sex-stratified estimates, widen uncertainty at the upper end of the exposure distribution, and complicate the interpretation of interaction results. Several biological mechanisms may also contribute to sex-specific differences. Estrogen may influence arterial wall remodeling by modulating collagen deposition and elastin production (35), and sex-related differences in nitric oxide-mediated endothelial function have also been reported (36). In women, especially during the menopausal transition and after menopause, changes in hormonal milieu, endothelial status, body fat distribution, uric acid metabolism, and HDL-C levels may alter the balance between uric acid-related oxidative stress and HDL-C-related vascular protection (37). In the present analysis, the female-specific association remained significant after additional adjustment for menopausal status, suggesting that menopausal status alone did not fully explain the observed pattern. Nevertheless, given the sparse representation of women in the higher UHR range and the exploratory nature of subgroup analyses, these results should not be interpreted as definitive evidence of sex-specific effect modification. Further studies with larger and more balanced sex-specific UHR distributions are needed to clarify whether sex truly modifies the association between UHR and baPWV.

From a practical standpoint, UHR has several appealing features. It can be obtained from routine biochemical testing, does not require additional cost, and is easy to calculate in large populations. In contrast to baPWV, which depends on dedicated equipment and trained operators. In medication-free health check-up populations, UHR may serve as a readily available marker associated with higher baPWV. That said, the present findings do not support using UHR as a replacement for direct vascular measurements. A more realistic role would be as an accessible indicator to identify individuals with higher baPWV who may warrant further vascular assessment.

This study has several limitations. First, the cross-sectional design precludes any inference about causality or temporal sequence. Therefore, the observed association between UHR and baPWV should be interpreted as correlational rather than causal. The present data cannot determine the temporal order between higher UHR and higher baPWV, or whether both are influenced by shared cardiometabolic factors. Second, all participants with current medication use were excluded. Although this restriction may have reduced confounding from pharmacological treatments, it also limited the generalizability of the findings to relatively healthy, medication-free individuals in a health check-up setting. Accordingly, the present results should not be directly generalized to patients receiving urate-lowering therapy, antihypertensive treatment, lipid-lowering therapy, glucose-lowering medication, or other long-term pharmacological treatments, because these medications may alter serum uric acid, lipid profiles, baPWV, or their association. Third, the source dataset overlaps with that used by Wang et al. (24). Therefore, the present study should be viewed as a focused reanalysis emphasizing sex-specific distribution and interpretation rather than as evidence from an independent population. Fourth, sex-specific analyses were limited by the markedly different distribution of UHR between men and women. Although menopausal status was additionally adjusted for in the female subgroup, women were sparsely represented in the higher UHR range, especially in the highest overall UHR quartile. Therefore, the sex-stratified findings should be interpreted cautiously and require confirmation in studies with larger female samples and more balanced UHR distributions. Fifth, residual confounding remains possible despite multivariable adjustment, particularly for factors that were not available in the dataset, such as inflammatory biomarkers and dietary patterns. Sixth, the participants were drawn from a Japanese health check-up population, which may limit the applicability of the findings to other ethnicities or clinical settings. Finally, arterial stiffness was assessed using baPWV only, and no comparison with other measures such as carotid-femoral pulse wave velocity was available.

## 5 Conclusion

In summary, among medication-free participants from a Japanese health check-up population, UHR was positively associated with baPWV independent of multiple confounding factors. The relationship did not appear to be strictly linear and may vary according to age and sex. The main added value of the present analysis is the focused evaluation of sex-specific patterns, which suggests that the female-specific association should be interpreted in light of marked sex differences in UHR distribution and sparse female representation in the higher UHR range. As a simple composite index derived from routine laboratory parameters, UHR may help identify medication-free health check-up participants who have higher baPWV and may warrant further vascular assessment. Prospective studies are needed to clarify the temporal relationship between UHR and changes in arterial stiffness and to determine whether UHR provides incremental value for cardiovascular risk assessment in clinical practice.

## Data Availability

The original contributions presented in the study are included in the article/**Supplementary Material**. Further inquiries can be directed to the corresponding author.
