# Supplementary figures and images for: Sex-specific distribution of the uric acid-to-HDL cholesterol ratio and its association with arterial stiffness in a Japanese health check-up population: a cross-sectional analysis
Source: Front Endocrinol (Lausanne). 2026 May 21;17:1847114. doi: 10.3389/fendo.2026.1847114 (PMC13233244; doi:10.3389/fendo.2026.1847114)

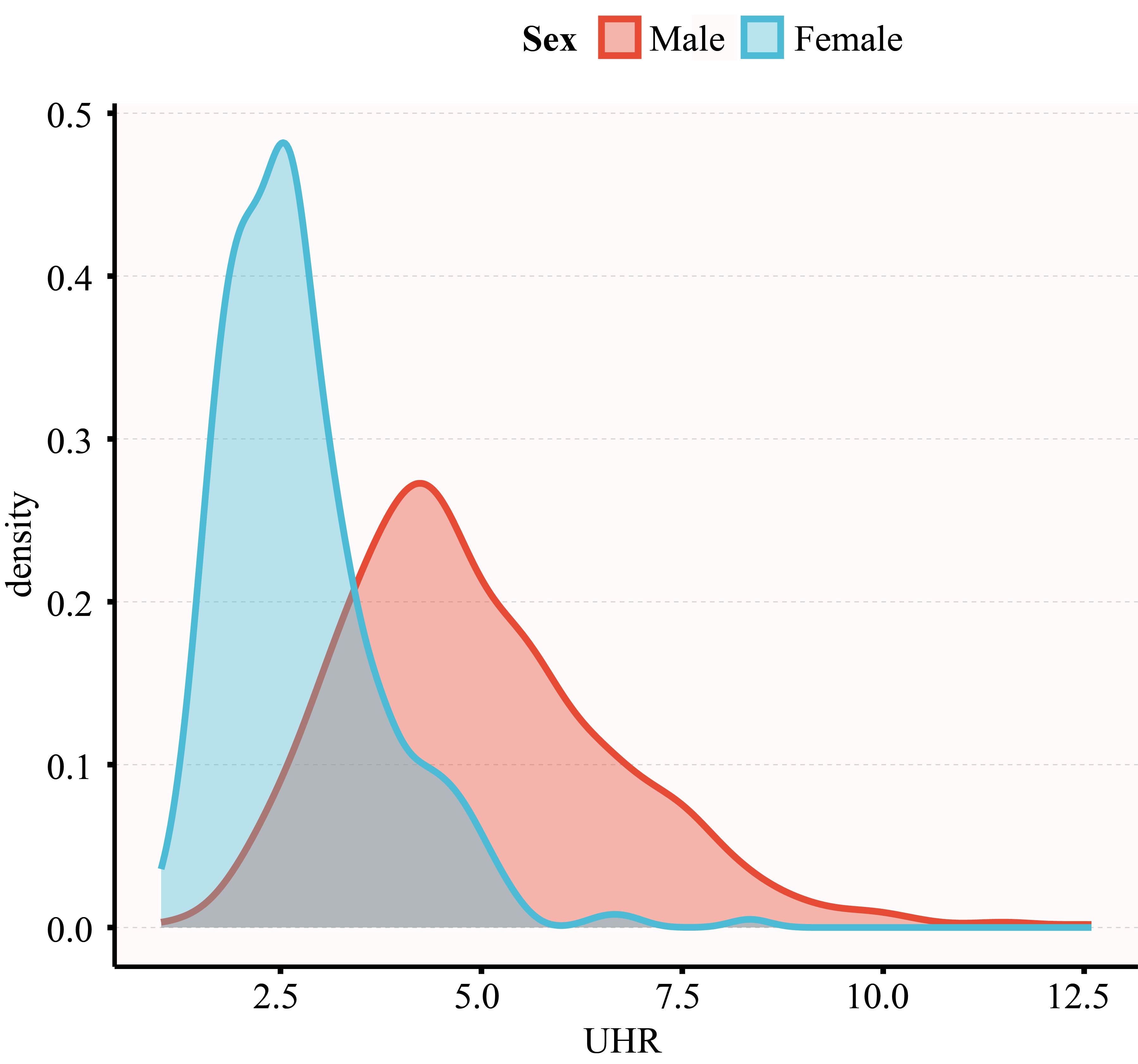

Supplement: Supplementary file 1 [file Image1.jpeg]
